# Physicochemical, Structural, and Nutritional Properties of Termite Mushroom-Fortified Tofu and Its Antioxidant Activity During In Vitro Digestion

**DOI:** 10.3390/foods15132295

**Published:** 2026-06-26

**Authors:** Nga Ngoc Quynh Nguyen, Hieu Tran-Van, Charles Brennan, Jayani Chandrapala, Thi Thu Hao Van

**Affiliations:** 1 School of Science, STEM College, RMIT University, Bundoora, VIC 3083, Australia; s3965877@student.rmit.edu.au (N.N.Q.N.); pvc.sciences@otago.ac.nz (C.B.); jayani.chandrapala@rmit.edu.au (J.C.); 2 Faculty of Biology and Biotechnology, University of Science, Vietnam National University, Ho Chi Minh 700000, Vietnam; tvhieu@hcmus.edu.vn; 3 Division of Sciences, University of Otago, Dunedin 9016, New Zealand; 4 Riddet Institute, Massey University, Palmerston North 4442, New Zealand

**Keywords:** termite mushroom powder, tofu, soy protein aggregation, minerals, β-glucan, antioxidant bioaccessibility, in vitro digestion, rheology, FT-IR

## Abstract

*Termitomyces albuminosus* is a wild edible mushroom with potential as a functional ingredient, yet its effect on tofu quality remains unclear. This study evaluated soy tofu fortified with *Termitomyces albuminosus* freeze-dried mushroom powder (TMP) at 1.5, 3, and 5% (*w*/*w*) using two strategies: direct addition and soybean replacement. The tofu treatments were assessed for yield, colour, texture, microstructure, molecular interactions, rheological behaviour, proximate composition, mineral profile, and antioxidant activity in fresh, cooked, and in vitro digested states. Increasing TMP progressively reduced yield, lightness, hardness, cohesiveness, and chewiness, with greater deterioration under high percentage replacement, associated with dose-dependent protein network coarsening and protein–polysaccharide phase separation; nevertheless, all samples retained viscoelastic gel behaviour (G′ > G″). The 1.5% replacement treatment largely preserved gel structure and texture, suggesting a favourable balance between enrichment and structural quality. The 5% replacement (R5) provided the greatest nutritional gain, significantly increasing calcium (2177.80 vs. 1812.43 mg/kg) and iron (27.07 vs. 20.61 mg/kg) compared to control while maintaining crude protein above 47% (dry basis). Antioxidant activity increased with TMP level and was highest in R5, with bioaccessibility peaking in the intestinal phase. TMP fortification represents a promising strategy for developing nutritionally enhanced tofu with improved mineral composition and antioxidant bioaccessibility.

## 1. Introduction

In recent years, heightened awareness about environmental sustainability and health-conscious dietary choices has elevated consumer interest in plant-based protein sources [1,2,3]. Among them, tofu, a semi-solid food formed by coagulating proteins in soymilk, has been a staple in vegan and vegetarian meals in Asia and an increasingly adopted product in Western and global markets [4,5]. It is positively perceived as a healthy, ethical and sustainable choice for meals [6]. Tofu is highly valued for its desirable nutritional profile of proteins, lipids, vitamins, minerals and antioxidants [7,8,9]. Additionally, tofu was reported to potentially reduce risk of diseases such as cardiovascular diseases [10], diabetes [11,12] and hyperlipidaemia [13,14]. Consequently, there have been considerable efforts to improve tofu quality and diversify product selections through multiple strategies including compositional modification, gelling condition optimisation and incorporation of functional ingredients [15,16,17].

Edible mushrooms have emerged as a promising source of functional food ingredients. Typically, mushrooms have low fat content of about 1–4% dry weight but they are rich in unsaturated fatty acids [18]. Their protein content is relatively high, accounting for approximately 15–40% dry weight in commercial species such as *Lentinus edodes*, *Agaricus* spp., and *Pleurotus* spp. with a complete profile of essential amino acids, making them a sustainable alternative to animal protein sources [19,20]. Alongside their compositions of minerals, B-group vitamins, and vitamin D, mushrooms also contain a range of bioactive compounds such as β-glucans, polyphenols, flavonoids, and terpenoids that exhibit potent antioxidant, immunomodulatory, antiviral, and anti-inflammatory activities [21,22,23,24,25]. Furthermore, edible mushrooms are palatable as they have an umami taste from glutamic acid, aspartic amino acids, and 5’-nucleotides [26,27]. Studies have shown that substitution of meat by mushrooms in meals was generally well received, satiable, and potentially induce weight loss in obese participants [28,29,30]. Therefore, edible mushrooms can be considered as a potential ingredient to fortify non-animal products such as tofu to enhance not only their nutritional value but also the taste and palatability. Moreover, mushroom cell walls contain derivatives of chitin [31], which can affect the gel properties and shelf life of tofu [32,33,34].

One of the most prized wild edible mushrooms, especially in Vietnam and Southern China markets, is white termite mushroom *(Termitomyces albuminosus)* or “Jizong” in China. It grows in a symbiotic relationship with termites during the rainy season and it is valued for meaty texture, sweet, fragrant flavour and the traditional beliefs in its medicinal values [35,36,37]. Similar to other common edible mushrooms, *Termitomyces* species contain 15–43% crude protein, 2–8% fat, 4–27% fibre (dry weight basis), and a range of minerals including Calcium, Iron, Magnesium, and Potassium [36]. Phenolic and polysaccharides extracts from *Termitomyces albuminosus* have exhibited antioxidant activities through reducing power, free-radical scavenging, and metal-chelating mechanisms, while also demonstrating hepatoprotective, antihyperlipidaemic, immunomodulatory, analgesic, and anti-inflammatory effects in both in vitro and in vivo experimental models [35,36,37,38,39,40,41]. These findings suggest *T. albuminosus* has potential as a functional food ingredient. However, current studies have largely focused on the bioactivities of extracts rather than evaluating *T. albuminosus* incorporation into real food matrices.

Research has investigated the integration of mushrooms into a range of food including bakery products (e.g., cereal bars, cookies, breads), dairy (e.g., yoghurts, cheeses), and meat alternatives (e.g., beef patty, pork sausages, chicken nuggets) to improve their nutritional and functional value [42,43,44]. Generally, additional mushroom powder or extracts from common mushrooms such as *Pleurotus* spp., *Flammulina* spp., and *Auricularia* spp. can enhance the antioxidant capacity of foods by improving phenolic content and radical scavenging and by reducing capacity [45]. Bread enriched with *Pleurotus eryngii and Cantharellus cibarius* powder had higher content of protein, fibre, lower glycaemic index, and enhanced aroma distribution but increased hardness and reduced fluffiness [46]. Additionally, positive effects of mushroom incorporation appear to be dose-dependent as bread texture, appearance, volume, and consumer acceptance decreased with more than 5% *Auricularia auricula* powder [47] or *Agaricus bisporus* powder [48]. Mushroom extracts showed enhanced probiotic viability in yoghurt but slightly lowered its sensory acceptance [49]. They also positively affected cheese quality, fibre content, and flavour [50]. By contrast, the application of mushrooms in tofu remains comparatively underexplored. To date, limited available studies have focused on the use of mushroom mycelia in tofu fermentation to promote flavour, texture, anti-cancer, and anti-inflammatory properties [51,52,53], whereas direct incorporation of whole mushroom powder into tofu formulations has been far less investigated.

The present study investigated the incorporation of whole freeze-dried *Termitomyces albuminosus* powder (TMP) into tofu and evaluated its effects on physicochemical properties, texture, rheological behaviour, nutritional composition, and antioxidant capacity in fresh, cooked, and simulated gastrointestinal-digested states. The novelty of this work lies in its direct use of whole TMP, rather than isolated extracts and in the systematic comparison of two incorporation strategies: direct supplementation and soybean substitution. By applying matched incorporation levels of 1.5%, 3%, and 5%, the study also enabled dose-dependent evaluation of the balance between nutritional enhancement and tofu gel quality. In addition, antioxidant activity was assessed across INFOGEST in vitro digestion, providing insight into the potential bioaccessibility of antioxidant compounds beyond conventional fresh or cooked product analysis. We hypothesised that TMP incorporation would enhance the mineral and antioxidant profile of tofu, while its non-gelling polysaccharide-rich fraction would progressively interfere with soy protein network formation, resulting in a formulation-dependent trade-off between functional enrichment and structural quality.

## 2. Materials and Methods

### 2.1. Tofu-Making Materials

Soybeans were purchased from Nut Grocer (Springvale, VIC, Australia) and calcium sulfate (gypsum) powder was obtained from Orku (Sumner, QLD, Australia).

Termite mushroom (*Termitomyces albuminosus*) was sourced from a local market in Ba Ria-Vung Tau, Vietnam. It was identified based on morphological characteristics by comparison with relevant taxonomic descriptions in the literature [54,55]. The mushroom was freeze-dried to an approximate moisture content of 9% and milled into fine powder using an ultra-centrifugal mill (ZM200, Retsch, Haan, Germany) fitted with 1 mm ring sieve, then stored in a sealed bag at room temperature.

### 2.2. Preparation of Tofu

**Figure 1:**
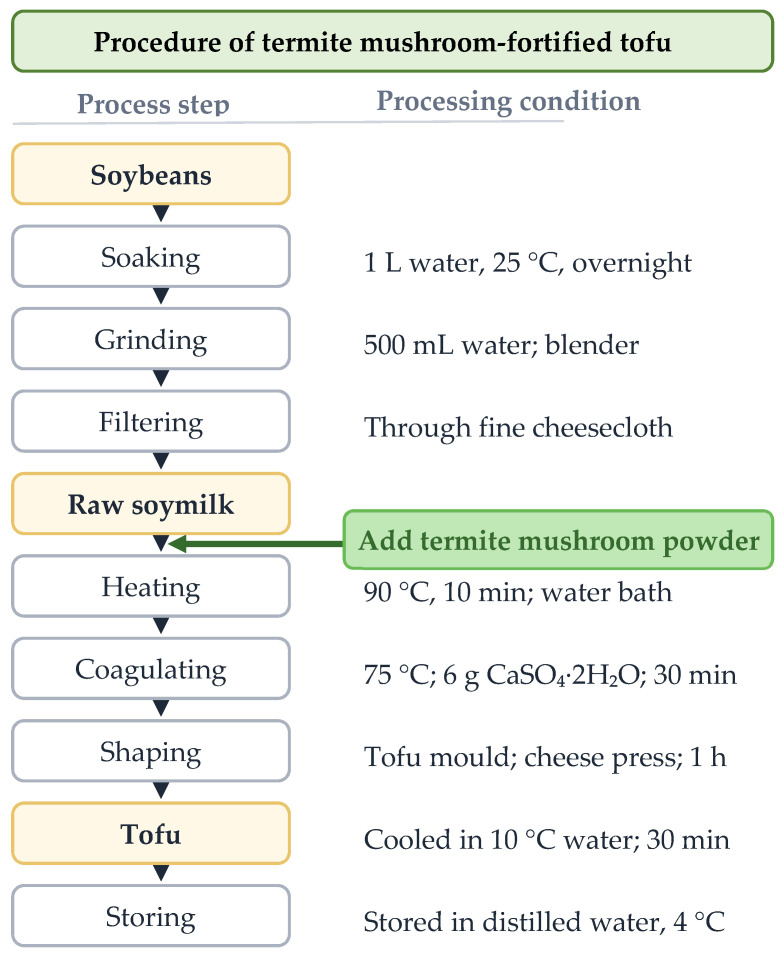
Procedure of tofu making.

The tofu preparation procedure was adapted from previously described methods [56,57] with minor modifications, as shown in Figure 1. There were seven treatments including control (150 g soybean), A1.5, A3, A5 (additional 1.5%, 3%, 5% *w*/*w* of termite mushroom powder (TMP), respectively), and R1.5, R3, R5 (1.5%, 3%, 5% soybean weight replaced by TMP, respectively). The TMP incorporation levels of 1.5%, 3%, and 5% were selected to represent low, intermediate, and high fortification levels within the technologically feasible range identified in preliminary trials, enabling dose–response evaluation while minimising severe disruption to tofu gel formation. The 5% upper limit was determined from preliminary trials, as TMP levels above 5% yielded structurally unstable curds unsuitable for quality analysis, consistent with previous reports of impaired texture and sensory acceptance at higher mushroom powder incorporation levels [47,48].

Soybeans were washed with tap water and soaked in 1 L of distilled water overnight at room temperature (25 °C). The soybeans were drained, then ground with 500 mL of distilled water at room temperature in a food blender (Nutri Ninja -Auto-iQ 1000, SharkNinja, Sydney, NSW, Australia) for 30 s. The slurry was filtered through fine-mesh cheesecloth to remove okara and obtain soymilk. TMP was added to soymilk and mixed well using the food blender for about 15 s.

The soymilk mixture was heated at 90 °C for 10 min using a water bath (Julabo TW 20, Seelbach, Germany). After being cooled down to 75 °C, 6 g of calcium sulfate (4% dry weight of soybean-mushroom mixture) was dissolved in 10 mL of distilled water at 75 °C and mixed well with soymilk for 10 s. The mixture was allowed to stand for 30 min at 75 °C to form curd which is then transferred to a 14 cm × 10 cm × 9 cm tofu mould (Mangocore, USA) lined with cheese cloth. The tofu is pressed for 1 h at room temperature using a cheese press.

Afterwards, the tofu was removed from the mould and weighed, then cooled down in 10 °C water for 30 min. The tofu was dipped in distilled water in a plastic box with lid and stored in a refrigerator (4 °C) overnight for further analysis. Tofu samples were prepared in three independent replicates. For each replicate, all seven treatments were produced within the same day, with subsequent measurements and analyses performed on the following day.

### 2.3. Physicochemical Quality Analysis

#### 2.3.1. Yield

Weight (g) of tofu after pressing was recorded. Wet-basis yield (%) was calculated as percentage of final tofu weight (g) over total weight of soybean and mushroom powder (g) used, as shown in Equation (1). Dry-basis yield (%) was calculated as dry weight of tofu (g) over total dry weight of ingredients (g), as in Equation (2). Dry weight was calculated as fresh weight minus moisture content.

(1) $$Yield_{wetbasis}(\%)=\frac{m_{tofu}}{m_{soybean}+m_{TMP}}\times 100\%\;$$

(2) $$Yield_{drybasis}\left(\%\right)=\frac{m_{tofu,dry}}{m_{soybean,dry\;}+m_{TMP,dry}}\times 100\%\;$$

#### 2.3.2. Moisture, pH, Water Activity, and Water Holding Capacity (WHC)

The moisture content (%) of tofu was assessed by a moisture analyser (Kern DBS, Kern & Sohn, Balingen, Germany). Five grams of tofu was cut into small cubes of 5 mm × 5 mm × 5 mm and dried at 105 °C until the sample reached a constant weight.

pH was measured by a pH meter (SevenCompact S220, Mettler Toledo, Columbus, OH, USA). Five grams of tofu was homogenised with 25 mL distilled water using a mortar and pestle, then filtered through cheesecloth to obtain the liquid for pH measurements [34].

Water activity (aw) of 5 g tofu was measured by a water activity meter (4TEV, Aqualab, Pullman, WA, USA).

Water holding capacity (WHC) was measured based on the method described previously [58]. Ten grams tofu was centrifuged (Sigma 3-30KS, Sigma Laborzentrifugen, Osterode, Germany) at 10,000× *g* at 25 °C for 10 min. The sample was then gently blotted with paper towels and reweighed. WHC was calculated as the percentage of the post-centrifugation weight (m_after_, g) relative to the initial weight (m_initial_, g), as in Equation (3).

(3) $$WHC\;\left(\%\right)=\frac{m_{after}}{m_{initial}}\times 100\%$$

#### 2.3.3. Colour Analysis

Colour analysis was conducted using a colorimeter (CR-400 Chroma Meter, Konica Minolta, Tokyo, Japan). The CIELAB colour parameters L*, a*, and b* were recorded, where L* represents lightness, a* represents the green-to-red axis, and b* represents the blue-to-yellow axis. Tofu cubes were placed directly under the optical glass of the instrument, and colour readings were taken at multiple locations.

### 2.4. Texture Profile Analysis

Texture profile analysis (TPA) was performed using a texture analyser (TA.XT Plus, Stable Micro Systems, Godalming, UK) following the methods of Hsieh et al. [57] with minor modifications. Tofu samples were cut into cubes (20 × 20 × 20 mm) and equilibrated at room temperature (25 °C) for 30 min prior to analysis. Each cube was subjected to a two-cycle compression test using a P/45 aluminium probe (45 mm diameter) to a final height of 10 mm (50% compression). The pre-test and post-test speeds were set at 2.0 mm/s, while the test speed was 0.5 mm/s. A 2 s interval was applied between the two compression cycles, with a trigger force of 5 g and a data acquisition rate of 10 points per second. The texture parameters recorded were hardness, springiness, cohesiveness, and chewiness.

### 2.5. Rheological Analysis

A rheometer (DHR 2, TA Instruments, New Castle, DE, USA) was used to observe the gelation process and viscoelastic properties of tofu samples based on methods described previously [57,59]. A plate geometry with a diameter of 40 mm was selected and the gap was set to 1 mm. The heat-treated soymilk mixture was stabilised at room temperature and mixed with calcium sulfate (4% total weight of soybean and mushroom) and immediately loaded onto the rheometer’s plate. The edge of the geometry was sealed with oil to prevent moisture loss.

The heat-treated soymilk mixture was stabilised at room temperature and mixed with calcium sulfate (4% dry bean and mushroom weight) and immediately loaded onto the rheometer’s plate. The sample was equilibrated at 25 °C for 5 min. To analyse the tofu making process, the temperature sweep was applied from 25 °C to 75 °C at the heating rate of 5 °C/min. Time sweep was performed at 75 °C for 30 min. The sample was cooled to 10 °C at the rate of 5 °C/min during a temperature sweep, then equilibrated at 10 °C for 30 min in a time sweep. Storage modulus (G′) and loss modulus (G″) were recorded every minute.

This was followed by a frequency sweep from 0.1–100 rad/s with the strain of 1% at 10 °C to investigate the rheological properties of tofu. TRIOS software (version 4.4) was utilised to process obtained data.

### 2.6. Confocal Laser Scanning Microscopy

The microstructure of the fresh tofu was observed using a confocal laser scanning microscope (C1 confocal microscope, Nikon Instrument Inc., Tokyo, Japan) with a 60× oil immersion objective (Plan Apo VC 60 × 1.40, Nikon Instrument Inc., Tokyo, Japan). Regarding dyes, Nile Red (1 mg mL^−1^) was prepared with dimethyl sulfoxide (DMSO) and Fast Green FCF (1 mg mL^−1^) with distilled water. Microscopy slides were prepared by cutting tofu into a thin slice (5 mm × 5 mm × 1 mm) using a surgical blade. Nile Red and Fast Green were each diluted to 0.1 mg mL^−1^. Nile Red was applied to the tofu section and allowed to air-dry for 30 min, followed by application of Fast Green and a further 30 min incubation. A coverslip was then placed and sealed with clear nail polish around the edges, and slides were analysed within 2 h of preparation. Nile Red and Fast Green FCF were excited using 488 nm and 561 nm laser lines, respectively, with emission filters selected at 500–530 nm for Nile Red and 568–643 nm for Fast Green FCF [60,61].

### 2.7. Fourier Transform Infrared Spectroscopy (FTIR) Study of Tofu Samples

Fourier transform infrared spectrometer (FTIR-GladiATR, PIKE technologies, Fitchburg, WI, USA) was used to scan thin tofu slices in the range of 400–4000 cm^−1^ with 16 scans and a resolution of 4 cm^−1^. Origin Software (Origin Pro, 2025; Origin Lab Corp, Northampton, MA, USA) was used to analyse spectra.

### 2.8. Nutritional Content

#### 2.8.1. Crude Protein Content

The crude protein content was assessed using a Kjeldahl digestion system connected to an auto analyser (KjeltecTM 8200 Auto Distillation Unit, FOSS, Hillerød, Denmark), following the Association of Official Analytical Chemists (AOAC) method [62]. Crude protein was calculated by multiplying the total nitrogen content by 6.25 [63].

#### 2.8.2. Ash Content and Mineral Profile

One gram of finely ground tofu sample was dried in a muffle furnace at 500 °C for 16 h [64]. Ash content was calculated as percentage of the weight of initial sample.

After ashing, 25 mL of 2% HNO_3_ was added to the sample, mixed well, diluted 100-fold with 2% HNO_3_ and filtered through a 0.45 μm filter before Inductively Coupled Plasma Mass Spectrometry (ICP-MS) mineral analysis, according to the method previously described by Pacquette, et al. [65]. An ICP-MS (7700 Series ICP-MS, Agilent, Santa Clara, CA, USA) coupled with an auto sampler (ASX-500 Series, Agilent, Santa Clara, CA, USA) was used to determine levels of macro and trace minerals (P, K, Ca, Na, Mg, Mn, Cu, Fe, Zn, Al). Calibration standards were prepared from an Agilent environmental calibration solution (part no. 5183-4688), which contained Fe, K, Ca, Na, and Mg at 1000 μg/mL each, and Ag, Al, As, Ba, Be, Cd, Co, Cr, Cu, Mn, Mo, Ni, Pb, Sb, Se, Th, Tl, U, V, and Zn at 10 μg/mL each. Standard for P was prepared in the concentration range of 0–10 mg/L.

### 2.9. Effects of Cooking

Tofu samples were prepared as previously described [66]; they were cut into cubes of 4 cm × 4 cm × 0.7 cm and fried in a deep fryer (F15, Roband, Cromer, NSW, Australia) at 130 °C for 3 min.

Cook loss (%) was calculated as percentage of weight loss after cooking over initial weight, according to Equation (4):

(4) $$Cook\;loss\;\left(\%\right)=\;\frac{m_{fresh}-m_{cooked}}{m_{fresh}}\;\times 100\%$$

Colour parameters L*, a*, b* of fried tofu were recorded by a colorimeter (CR-400 Chroma Meter, Konica Minolta, Japan) at least six times. ΔE was calculated following Equation (5) to monitor the change after cooking.

(5) $$∆E=\sqrt{{\left(L_{cooked}^{*}-L_{fresh}^{*}\right)}^{2}+{\left(a_{cooked}^{*}-a_{fresh}^{*}\right)}^{2}+{\left(b_{cooked}^{*}-b_{fresh}^{*}\right)}^{2}}$$

### 2.10. In Vitro Digestion

In vitro digestion of cooked tofu was based on the INFOGEST 2.0 model [67] including oral, gastric, and intestinal digestion phases. The cooked tofu sample was crushed by a grinder (MultiGrinderTM-EM0405, Sunbeam, Botany, NSW, Australia) for 20 s.

Oral phase: 5 g of ground cooked tofu was mixed well with simulated saliva fluid, salivary amylase, and distilled water and was incubated while mixing for 2 min at 37 °C in a shaking water bath (Grant OLS26, Grant instrument, Beaver Falls, PA, USA).

Gastric phase: the bolus from oral phase was mixed with simulated gastric fluid and pepsin, with pH adjusted to 3.0 using 1M HCl and incubated while mixing at 37 °C for 2 h.

Intestinal phase: the gastric chyme was mixed well with simulated intestinal fluid, pancreatin and bile salt. The pH was adjusted to pH 7.0 using 1M NaOH. The mixture was incubated while mixing at 37 °C for 2 h.

Digestive supernatants were collected at the end of each phase and heated at 95 °C for 5 min to inactivate enzymatic activities [68]. The mixture was then centrifuged at 2500× *g* for 15 min at 25 °C to collect the supernatant which was filtered through Whatman Filter Paper Grade 1 before being stored at −80 °C for further analysis.

### 2.11. Measurement of Antioxidant Potential

The antioxidant activities of each sample and its digested supernatants were measured by total phenolic content, DPPH, ABTS, and FRAP.

#### 2.11.1. Extraction of Phenolic Compounds

The extraction from soybean, termite mushroom powder, fresh, and cooked tofu followed the protocol stated by Gogoi, et al. [69] with slight modifications. Ethanol solvent was added to each sample at the ratio of 20 mL/g and treated with ultrasound frequency of 40 kHz, ultrasonic power 100 W for 26 min at 39 °C using an ultrasonic bath (FXP12, Unisonics, Brookvale, NSW, Australia). The mixture was centrifuged at 2500× *g* for 15 min at room temperature. The supernatant was filtered through Whatman filter paper grade 1 and stored at −80 °C.

#### 2.11.2. Total Phenolic Content

To quantify the total phenolic content in the extraction of soybean, termite mushroom powder, fresh tofu, cooked tofu, and collected digesta, the Folin–Ciocalteu method was applied using a 96-well microplate according to previously described methods [70,71] with modifications.

Gallic acid was used as the standard at concentrations of 0, 40, 80, 120, 160, 200 mg/L. Ten microlitres of standard or sample was added to each well in triplicate, then 10 μL of Folin–Ciocalteu reagent (2 N) was added, mixed well, and left to stand for approximately 8 min. Then, 30 μL of 20% (*w*/*v*) of Na_2_CO_3_ and 150 μL of distilled water was added to each well to make a total volume of 200 μL. The plate was incubated in darkness at 37 °C for 30 min, shaken for 300 s and read absorbance at 765 nm using a microplate reader (CLARIOstar Plate Reader, BMG Labtech, Ortenberg, Germany).

#### 2.11.3. Ferric Reducing Antioxidant Power (FRAP) Assay

Acetate buffer (0.3 M pH 3.6), TPTZ solution (10 mM dissolved in 40 mM HCl), and FeCl_3_ solution (20 mM) were mixed according to the ratio 10:1:1 to make the FRAP working solution. Trolox was used as the standard at concentration 10, 20, 40, 60, 80, 100, 200 µM in ethanol. The procedure of the assay followed the published method [72,73] with slight modifications.

Twenty microlitres of standard or sample were added to each well of the microplate, followed by 280 µL of working FRAP solution and mixed well. The plate was incubated at 37 °C for 30 min and the absorbance was recorded at a wavelength of 593 nm using a CLARIOstar microplate reader.

#### 2.11.4. ABTS Assay

ABTS assay followed the procedure described by Xiao, et al. [74]. ABTS (2,2′-azino-bis(3-ethylbenzothiazoline-6-sulfonic acid)) stock solution was made at 7 mM in acetic acid buffer (pH 4.5) and Potassium persulfate (K_2_S_2_O_8_) at 2.45 mM. They were mixed at 1:1 ratio and kept at room temperature overnight to form ABTS reaction solution, which was further diluted with acetic acid buffer to achieve absorbance at 734 nm at 0.74 ± 0.03.

Two hundred microlitres of working reagent was added to 10 µL of sample in a 96-well plate. Following a 7-min incubation in the dark at room temperature, the absorbance was recorded at 734 nm using a CLARIOstar microplate reader. Trolox standard curve (blanked with distilled water) was used. All sample and standard readings were kept between 0.2 and 0.8 absorbance units through appropriate dilution.

#### 2.11.5. DPPH Assay

The DPPH radical scavenging assay was conducted following the established methods [75,76] with minor modifications. A 0.125 mM DPPH (2,2-diphenyl-1-picrylhydrazyl) was prepared in ethanol and adjusted to obtain an absorbance reading of 0.8–1.0. Trolox was used as the standard with concentration ranging from 10 to 200 µM in ethanol.

On a 96-well microplate, 100 µL of samples or standard was mixed well with 100 µL of DPPH working solution and incubated in the dark at room temperature for 30 min. The absorbance was then read at 517 nm using a microplate reader (CLARIOstar Plate Reader, BMG Labtech, Ortenberg, Germany). Ethanol was used as blank. Antioxidant capacity was quantified using a Trolox calibration curve with results expressed as µmol Trolox equivalents antioxidant capacity per gram (µmol TEAC/g).

### 2.12. Statistical Analysis

IBM-SPSS software version 31 (SPSS Inc., Chicago, IL, USA) was used to conduct variance analysis and Tukey’s post hoc test at a significance level of *p* < 0.05. OriginPro 2025 software (OriginLab Corporation, Northampton, MA, USA) was used for graphing and FT-IR analysis. All standards and samples were measured in triplicate, unless otherwise stated. Data are presented as the mean ± standard deviation (SD).

## 3. Results

### 3.1. Tofu Yield and Quality Characteristics

The yield and physicochemical quality characteristics of control and termite mushroom powder (TMP)-fortified tofu are presented in Table 1.

Statistically significant differences among the treatments (*p* < 0.05) were observed for wet-basis and dry-basis yield and water holding capacity, while no significant differences were found for moisture, pH, water activity, and cook loss.

Yield was highest in the control sample (177.53 ± 16.97% wet basis; 50.75 ± 0.25% dry basis) and generally decreased with TMP incorporation in both the addition and replacement series. Yields did not differ significantly between the addition and replacement series; however, replacement at the lower level retained relatively higher yields than the corresponding addition treatments. Among TMP treatments, A3 exhibited the lowest wet-basis yield (115.00 ± 5.08%), while A5 showed the lowest dry-basis yield (34.60 ± 6.10%); both were significantly lower than the control.

Water holding capacity (WHC) exhibited a non-linear pattern across the treatments. R1.5 achieved the highest WHC (87.80 ± 2.90%) while R5 had the lowest value (72.02 ± 2.19%). The other TMP treatments maintained WHC values comparable to the control (80.08 ± 4.77%).

pH stayed in a neutral range (6.43 ± 0.10) and water activity values were near unity (a_w_ ≈ 1.00), which is typical for fresh tofu and consistent with their high moisture content (73.21 ± 2.53%) [77,78]. Despite differences in WHC, cook loss remained similar across treatments (25.80 ± 3.99%).

### 3.2. Colour Analysis

Table 2 shows colour parameters L*, a*, b* before and after cooking and the colour changes induced by cooking.

For each colour, two-way ANOVA analyses showed there are significant differences due to treatment (*p* < 0.05), cook status (*p* < 0.001), and interaction between cook status and treatment (*p* < 0.001). For L*, the control was the brightest whereas R5 was the darkest in both states. Cooking decreased brightness and the effect was generally more evident with increased content of TMP. The change was most evident in A3 treatment.

For a*, fresh control was most neutral whereas TMP tofu, especially A3 and R3, had more red tone. Both higher TMP percentage and cooking increased redness, which was most obvious in A3 treatment. For b*, cooking clearly enhanced yellowness of samples, especially in A1.5 and A3 samples.

ΔE value is greater than 5 in all treatments, indicating that the changes of colour were visible and A3 showed the most dramatic shift (15.26) while it was least intensive in control treatment (7.04).

### 3.3. Proximate Nutritional Content

The protein content, ash, and mineral profiles of all tofu treatments are presented in Table 3.

Protein content (dry basis) showed a tendency to decrease with increasing TMP concentration, declining from 57.79 ± 5.94% in control to 47.81 ± 1.87% in A1.5 and 48.24 ± 5.83% in R5 (*p* < 0.05). Ash content did not show significant differences between treatments, suggesting that the total inorganic content remained relatively constant.

Among macrominerals, calcium was the most abundant element across all treatments (1603.25–2177.80 mg/kg d.b.), reflecting the use of CaSO_4_ as a coagulant. Notably, R5 exhibited the highest Ca content (2177.80 ± 174.71 mg/kg), significantly greater than A1.5 (1603.25 ± 121.79 mg/kg, the lowest value) and the control (1812.43 ± 146.70 mg/kg) (*p* < 0.05). Phosphorus and magnesium contents showed no significant differences among treatments (*p* > 0.05), though P showed a numerical upward trend from A1.5 (830.99 mg/kg) to A5 (1053.61 mg/kg). Potassium content differed significantly (*p* < 0.05), with R3 (500.50 ± 53.35 mg/kg) being significantly higher than A1.5 (287.93 ± 28.01 mg/kg), while other treatments fell within intermediate groupings.

Sodium content showed a non-linear treatment effect. Control (852.47 ± 112.30 mg/kg) and A1.5 (888.82 ± 69.72 mg/kg) had significantly higher Na than all other treatments (*p* < 0.001). However, R5 occupied an intermediate position and the rest clustered at lower levels.

Among trace minerals, iron showed the clearest dose-dependent response to TMP fortification. R5 (27.07 ± 7.14 mg/kg) was significantly higher than the control (20.61 ± 2.65 mg/kg) (*p* < 0.05). Zinc, manganese, and copper contents remained statistically unchanged across all treatments (*p* > 0.05), though Zn showed a modest numerical increase at higher TMP levels (Control: 9.90 vs. R5: 11.59 mg/kg). Aluminium content also varied, with the control and A1.5 treatment showing higher values than others.

Overall, although there was not a consistent trend across the minerals examined, R5 had the highest values for most minerals.

### 3.4. Texture Profile Analysis

TPA parameters of tofu samples are presented in Table 4.

The control sample exhibited the highest hardness and cohesiveness, which translated into the greatest chewiness. Increasing the concentration of TMP generally reduced hardness, cohesiveness, and chewiness relative to the control. Among the TMP-fortified samples, A3 showed the highest hardness and chewiness, although these remained below the control. The addition treatments retained texture parameters better than the replacement treatments.

For the R series, R1.5 retained relatively high hardness and chewiness. However, R3 and R5 showed marked reductions in hardness and chewiness, with values not significantly different from each other.

Springiness (ratio) remained close to unity across all treatments (range: 0.99917–0.99950), with only minor but statistically significant differences detected among groups, indicating that the elastic recovery of the samples was largely unaffected by the treatments.

### 3.5. Confocal Laser Scanning Microscopy (CLSM)

**Figure 2:**
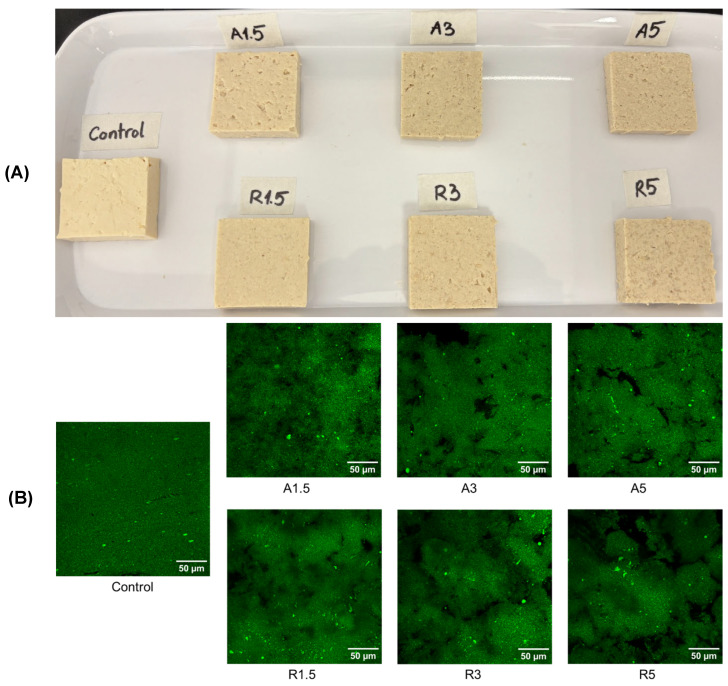
Macrostructure appearance of TMP-fortified tofu (**A**) and CLSM images of tofu microstructure with a 60× objective; green colour indicating protein dyed by Fast Green FCF (**B**).

Figure 2 shows the structure of tofu treatments. Figure 2A showed fresh cut surfaces of tofu treatments. The control sample had a smooth and even surface whereas TMP-fortified tofu had visible pores. This is consistent with confocal laser scanning microscopy (CLSM) images in Figure 2B, which revealed notable differences in the protein network microstructure of tofu samples across treatments. The control sample exhibited a relatively homogeneous and dense protein matrix with uniformly distributed fine protein aggregates, indicating a well-formed, continuous gel network. In contrast, both the A (1.5, 3, 5) and R (1.5, 3, 5) treatment series showed progressively coarser and more heterogeneous microstructures with increasing TMP concentration. Dark voids, corresponding to water-filled cavities or serum channels, became larger and more irregularly distributed as the treatment level increased, suggesting disruption or reorganisation of the soy protein network.

Comparing the A and R series, at lower TMP percentage, R1.5 showed a relatively more even surface (Figure 2A) and denser protein network (Figure 2B) than A1.5. However, at higher TMP level, the R series displayed a more pronounced coarsening of the gel matrix with larger pores and more extensive phase separation, particularly evident at R3 and R5. Overall, the microstructural changes are consistent with a dose-dependent weakening of the tofu gel network.

### 3.6. FT-IR Spectra

**Figure 3:**
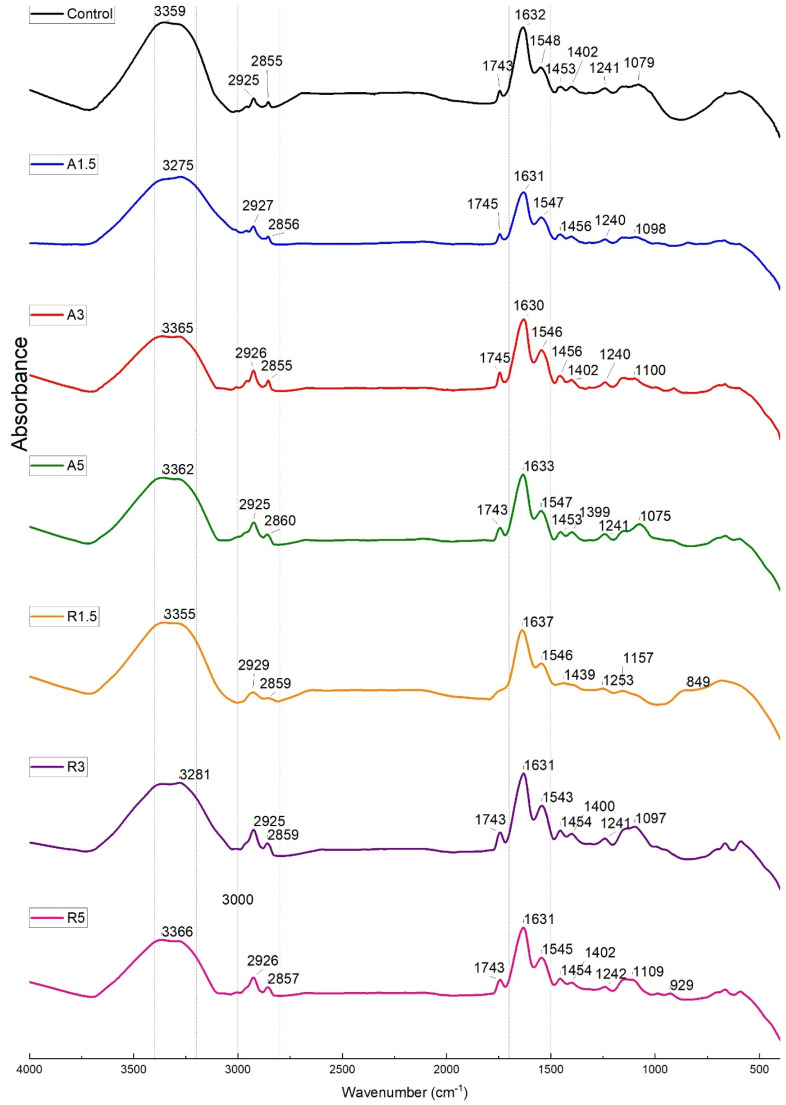
FT-IR spectra of tofu control and TMP treatments.

Figure 3 shows the FT-IR spectra of control and TMP-fortified tofu samples. All spectra exhibited characteristic protein absorption bands of tofu: a broad band at O–H/N–H stretching region (3000–3600 cm^−1^), amide I band at approximately 1630–1660 cm^−1^ (primarily C=O stretching), amide II band at approximately 1530–1550 cm^−1^ (N–H bending coupled with C–N stretching), and amide III band near 1230–1240 cm^−1^ [79]. A band at ~1743 cm^−1^ in all treatments (except R1.5) confirmed C=O ester stretching of soy triglycerides.

In O–H/N–H stretching region, control peaked at 3359 cm^−1^, nearly identical to R1.5. While A1.5 (3275 cm^−1^) and R3 (3281 cm^−1^) showed a pronounced downshift, A3, A5, and R5 showed peaks at higher wavenumber (3362–3366 cm^−1^). Similarly, changes in the amide I region were observed. The control sample showed an amide I peak at 1632 cm^−1^. In the addition series, the peak shifted slightly to 1631 cm^−1^ in A1.5 and 1630 cm^−1^ in A3, then to 1633 cm^−1^ in A5. In the replacement series, R1.5 showed the highest amide I wavenumber at 1637 cm^−1^, whereas R3 and R5 both exhibited peaks at 1631 cm^−1^.

Variation among treatments was evident in the polysaccharide fingerprint region (800–1200 cm^−1^). All samples showed bands at 1075–1109 cm^−1^. R1.5 exhibited an additional band at 849 cm^−1^ and a distinct absorption at 1157 cm^−1^, while R5 showed a unique band at 929 cm^−1^. Compared with the other treatments, R1.5 also lacked the ~1743 cm^−1^ band corresponding to C=O ester stretching of triglycerides existing in other samples.

### 3.7. Rheology Properties

#### 3.7.1. Initial Temperature Sweep

**Figure 4:**
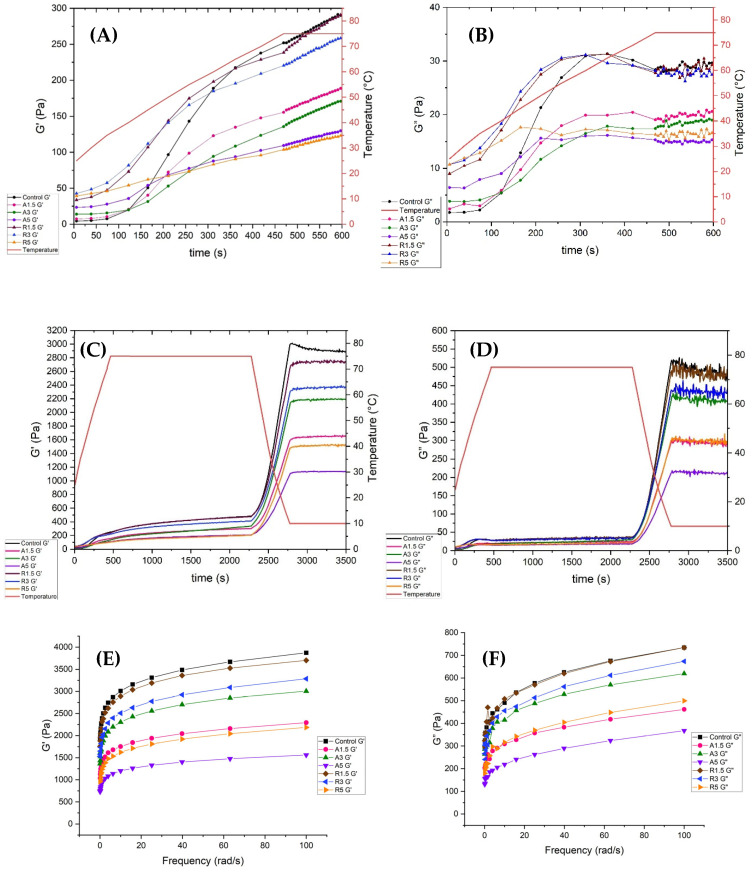
Changes of storage (G′) and loss (G″) modulus of TMP-enriched soymilk after calcium sulfate addition during the first time sweep 25–75 °C (**A**,**B**), through the temperature change of the whole tofu making process (**C**,**D**) and during frequency sweep (1–100 rad/s) at 10 °C (**E**,**F**).

The evolution of storage modulus (G’) and loss modulus (G”) during the initial temperature sweep from 25 to 75 °C is presented in Figure 4A,B. From the onset of measurement (t = 0 s), G′ exceeded G″ for all treatments, so there was an immediate elastic network formation prior to thermal treatment. Notably, replacement treatments R5 and R3 exhibited higher initial G′ values (~40–50 Pa) compared with the control (~5 Pa). All TMP treatments showed a significantly lower initial tanδ (~0.25–0.31) (*p* < 0.05) compared to control (0.48 ± 0.09).

When the temperature started to rise, R1.5 and R3 displayed the fastest rate of G′ increase while others’ G’ increased slightly. When temperature progressed beyond 50 °C, the control’s G′ became the highest at approximately 60 °C and continued to develop the highest modulus throughout the heating phase. At 75 °C, the highest G’ belonged to control (~280 Pa), followed closely by R1.5 and R3 while A1.5 and A3 stayed in the intermediate group. Interestingly, A5 and R5 showed the smallest G’ increase, which followed an approximately linear trend. The G″ values followed similar trends but at substantially lower magnitudes (maximum ≈30 Pa), confirming predominantly elastic gel character throughout the heating phase.

#### 3.7.2. Complete Gelation Profile

The complete gelation profile encompassing heating (25–75 °C), isothermal holding (75 °C, 30 min), cooling (75–10 °C), and isothermal holding at 10 °C is shown in Figure 4C,D. During the 30 min isothermal hold at 75 °C, G′ continued to increase gradually for all treatments, with control and R1.5 maintaining the highest G′ (≈450 Pa). The remaining treatments plateaued at 100–350 Pa, with A5 and R5 exhibiting the lowest modulus (≈100 Pa).

Upon cooling from 75 to 10 °C, all treatments exhibited a dramatic and rapid increase in both G′ and G″. The magnitude of this cooling-induced gel strengthening was treatment-dependent although the ranking remained unchanged from the previous phase. The control achieved the highest final G′ (≈3000 Pa), followed by R1.5 (≈2700 Pa), R3 (≈2350 Pa), A3 (≈2150 Pa), A1.5 (≈1650 Pa), R5 (≈1500 Pa), and A5 (≈1050 Pa). The G″ values increased in parallel but to a lesser extent, reaching approximately 500 Pa (Control), 500 Pa (R1.5), 420 Pa (R3 and A3), 300 Pa (R5 and A1.5), and 210 Pa (A5). The ratio G′/G″ therefore increased upon cooling for all treatments, indicating that cooling enhanced the elastic character of the gels disproportionately relative to the viscous component. During the subsequent isothermal hold at 10 °C, both G′ and G″ stabilised, confirming the formation of equilibrium gel networks.

#### 3.7.3. Frequency Sweep

Frequency sweep data for the final gels at 10 °C are presented in Figure 4E,F. G′ was substantially greater than G″ across the entire frequency range (1–100 rad/s), confirming true gel behaviour. Both G′ and G″ showed moderate frequency dependence, increasing with angular frequency. The ranking of amplitude of both G’ and G” were similar to previous phases.

### 3.8. Antioxidant Activities

Table 5 presents the results of antioxidant assays—total phenolic content (TPC), DPPH, ABTS and FRAP—of tofu treatments across fresh and cooked states and at the end of three phases of in vitro digestion including oral, gastric and intestinal phases.

Overall, the cooked state exhibited higher antioxidant activity than the fresh state in all assays except DPPH. In addition, the final intestinal phase showed a marked increase in antioxidant activities for all samples.

In fresh samples, TMP fortification significantly increased total phenolic content (TPC) and ABTS radical scavenging activity relative to the control (*p* < 0.05), whereas FRAP and DPPH result remained low and did not differ significantly among treatments. Cooking significantly increased TPC and ABTS results in all treatments and increased FRAP in most samples, while DPPH showed no significant differences between fresh and cooked states. Among the cooked samples, R1.5 exhibited the highest TPC (8.16 ± 0.46 mg GAE/g), R5 showed the highest ABTS value in the fresh state (4.77 ± 0.15 µmol TEAC/g), and A1.5 recorded the highest FRAP value in the cooked state (2.83 ± 0.28 µmol TEAC/g).

Simulated digestion markedly altered antioxidant behaviour. TPC was low in the oral phase (0.63–0.82 mg GAE/g), increased in the gastric phase (1.49–2.17 mg GAE/g), and reached its highest values in the intestinal phase (3.44–4.82 mg GAE/g), following a consistent oral < gastric < intestinal trend across all treatments (*p* < 0.05). Similarly, DPPH activity generally increased during digestion, particularly in the gastric phase (6.49–13.60 µmol TEAC/g), and showed strong treatment dependence in the intestinal phase. R1.5 exhibited the highest intestinal DPPH value (29.44 ± 2.06 µmol TEAC/g) but A1.5 and R5 remained comparatively low. ABTS showed the clearest and most consistent phase-dependent increase, rising sharply during digestion and reaching uniformly high values in the intestinal phase (140.49–169.80 µmol TEAC/g), with no significant differences among treatments though R5 still showed the highest value (169.8 ± 1.75 µmol TEAC/g). FRAP displayed a similar but less pronounced pattern, remaining low in the oral and gastric phases (0.50–0.85 µmol TEAC/g) before increasing markedly in the intestinal phase (8.84–12.07 µmol TEAC/g), again without significant treatment differences. Overall, the intestinal digestion phase was the principal stage at which antioxidant compounds became bioaccessible.

## 4. Discussion

### 4.1. Changes in Chemical Structure During Tofu Formation and Effects of Termite Mushroom Powder

#### 4.1.1. Soybean Proteins During Tofu Formation

Protein is a principal constituent of soybean, accounting for about 40% of its dry weight composition [80,81]. Soybean proteins include approximately 90% globulins and 10% albumins. Within the globulin fraction, 11S (glycinin) and 7S (β-conglycinin, β-amylase, lipoxygenases, and lectins) represent approximately 42% and 34%, respectively [82,83,84]. Their ratio and composition strongly influence tofu quality attributes such as texture, yield, water holding capacity [85,86].

At room temperature, proteins in raw soymilk remain relatively stable as the hydrophobic regions remain inside the protein molecule while hydrophilic groups are on the surface, exposed to the aqueous environment [81]. During heating, increased molecular motion disrupts secondary bonds that maintain protein conformation, leading to subunit dissociation, unfolding, and changes in the three-dimensional structure. Consequently, reactive hydrophobic sites such as sulfhydryl groups, disulfide bonds, amino acid side chains become exposed, promoting protein–protein interactions and aggregation [87]. Denaturation temperature of 7S protein (~70 °C) is lower than that of 11S protein (~80 °C) [88]. When the temperature reaches 80–100 °C, solubility of the globulins decreases and their α-helical structure gradually converts into β-sheet, then to β-turns and random coil structures, which are essential to the formation of aggregates [89]. This structural endpoint was evident in the FT-IR spectrum of control tofu in Figure 3. The amide I band at 1632 cm^−1^ falls within the 1620–1640 cm^−1^ window diagnostic of β-sheet conformation [90], indicating that heat-induced denaturation of the soymilk globulins followed by aggregation drove the protein toward an intermolecular β-sheet-rich gel network. The accompanying amide II band at 1548 cm^−1^ and amide III band at 1241 cm^−1^ reflect coupled C–N stretching and N–H bending of the peptide backbone [91,92].

At lower degrees of denaturation, hydrophobic interactions have been reported to be the main protein–protein interaction to form a gel structure, and disulfide bonds become more dominant at higher degrees of denaturation [93]. After calcium sulfate salt addition, released Ca^2+^ cations neutralise negative-charged protein surfaces and decrease electrostatic repulsion, allowing molecules to move closer. Ca^2+^ cations form salt bridges between adjacent protein molecules by ionic bonds with proteins’ carboxyl groups, promoting protein aggregation into a continuous three-dimensional tofu gel network [94,95]. Consistent with this mechanism, the control sample’s band at 1402 cm^−1^ was associated with carboxylate (COO^−^) stretching, indicating the typical ionised protein side chains involved in calcium-mediated cross-linking [96]

The hydrated protein-rich network of control tofu was also reflected by the broad band at 3359 cm^−1^, which is attributed to the overlapping O–H stretching vibrations of water and hydroxyl groups, together with the amide A N–H stretching mode of the peptide backbone [97]. The bands at 2925 and 2855 cm^−1^ (in 2850–2960 cm^−1^ region) correspond to symmetric and asymmetric C–H stretching vibrations of methylene (–CH_2_–) and methyl (-CH_3_) groups from aliphatic amino-acid side chains of the soy protein or from triglyceride acyl chains of soybean oil. Additionally, from soybean oil, the band at 1743 cm^−1^ is assigned to the ester carbonyl (C=O) stretching of triglycerides. Lastly, C–O/C–O–C stretching from soybean carbohydrates was observed at 1079 cm^−1^ [91].

#### 4.1.2. FT-IR Spectra of Tofu and the Incorporation of Termite Mushroom

*Termitomyces* species are rich in protein with crude content accounting for about 20–40% dry weight. They also contain high levels of carbohydrate, ranging from 25% to a high of nearly 60% dry weight [36]. In *Termitomyces albuminosus*, approximately 25% of the dry matter is contributed by polysaccharides, which is considered among the major bioactive constituents of edible mushrooms [37]. Hong and Ying [98] reported that *T. albuminosus* has a high yield (13.46% dry weight) of chitin–glucan complex which contains β-D-glucan and chitin in a molar ratio of 46:54. Thus, the incorporation of TMP would introduce an additional polysaccharide-rich portion to tofu’s protein matrix.

FT-IR spectra provide molecular-level information on chemical bonds and functional groups in different TMP-tofu treatments. As seen in Figure 3, the major bands were preserved across the treatments indicating that TMP incorporation did not fundamentally disrupt the characteristic soy protein–lipid gel matrix of tofu. In particular, the amide I band (1600 to 1700 cm^−1^ region) remained within 1630–1637 cm^−1^ across treatments, which is consistent with the β-sheet-rich secondary structure (1610–1640 cm^−1^) commonly reported for soy storage proteins glycinin and β-conglycinin [96,99]. β-sheets are formed by parallel or antiparallel polypeptide chains that are stabilised by hydrogen bonding, maintaining a compact protein conformation of tofu structure [99]. Compared to the control, the absence of new absorption bands in TMP treatments hinted that no major new covalent bonds or chemical structures were formed between the soybean protein and mushroom polysaccharide, reflecting that interactions between soymilk and TMP were likely dominated by non-covalent forces [100].

However, concentration-dependent changes were observed. Shifts in the 3000–3600 cm^−1^ region (O–H bond, N–H bond) are established indicators of hydrogen bonding strength, as lower stretching frequencies correlate with stronger hydrogen bonds and shorter O–H···O distances [101,102]. The downshift at low TMP levels (A1.5, R1.5) relative to the control indicates strengthened hydrogen bonding, consistent with favourable integration of β-glucan hydroxyl groups into the protein–water hydrogen bonding network [103]. At this level, the abundant hydroxyl groups of β-glucan can insert cooperatively into the protein matrix by forming hydrogen bonds with exposed polar side chains of denatured soy protein, bridging through interfacial water [104,105]. In contrast, at higher TMP concentrations, the O–H/N–H peaks shifted to slightly higher wavenumbers (3362–3366 cm^−1^), indicating modest weakening of the hydrogen bonding environment [106], likely associated with increasing thermodynamic incompatibility between the protein matrix and mushroom polysaccharides. This non-linear pattern shows a transition from cooperative protein–polysaccharide interaction at low concentrations to disruption of hydrogen bonds at higher polysaccharide loads.

Treatment-dependent shifts in the amide I region (1600 to 1700 cm^−1^) indicate that TMP affected protein molecular organisation. Because the amide I band (assigned to C=O vibrations) is highly sensitive to protein secondary structure and hydrogen bonding within the peptide backbone, even small shifts can reflect conformational rearrangement [107]. In the addition series, upshift in A5 may indicate reduced structural order at the highest TMP addition level while the slight downshifts observed in others (A1.5, A3) may suggest subtle strengthening of soy protein’s β-sheet networks induced by protein–polysaccharide co-aggregation [103]. In a protein–polysaccharide system, polysaccharides can bring protein molecules to closer contact, promoting aggregation to form β-sheet structure at moderate level due to electrostatic interactions. However, increasing polysaccharide content beyond a compatible level can dilute the effective gelling protein components (e.g., concentration of 7S and 11S in soy protein) and alter electrostatic interactions, shifting the system to phase separation [108]. For instance, mostly neutral β-glucan does not attract negatively charged groups on protein, so the two biopolymers become incompatible and separate into protein-rich and polysaccharide-rich domains, disrupting the continuity of the soy protein gel network. Similar non-linear effects of polysaccharides on soy protein gel structure have been reported previously, with moderate levels promoting stabilisation but higher levels leading to partial disruption of ordered arrangements [100]. On the contrary, the replacement series did not show a clear trend as the band downshifted at higher concentration (R3, R5—1631 cm^−1^) and upshifted at low concentration (R1.5—1637 cm^−1^). This contrast indicates that the addition series primarily reflects the concentration-dependent effect of TMP on a constant soy protein matrix, whereas the replacement series reflects the combined effects of protein dilution and polysaccharide incorporation, leading to a more complex and less predictable structural response.

The absence of peaks in regions of α-helix (1648–1664 cm^−1^), β-turn (1664–1681 cm^−1^) and random coil (1637–1648 cm^−1^) showed that β-sheets were the dominant protein secondary structure across treatments, and the α-helix structure was most probably denatured during heat treatment [109]. At around 1245 to 1550 cm^−1^, amide II and III bands were determined [79]. The region between 1200 to 1400 cm^−1^ typically presents vibrations of CH_2_, C–C, C–O, and C–H groups [110]. In the region 800–1200 cm−^1^, the peaks within the polysaccharide fingerprint region also confirmed the progressive integration of TMP polysaccharides into the gel matrix, with variation in peak position indicating differences in the local polysaccharide environment. Bands at around 1065–1110 cm^−1^ are characteristic of β-glucans [111]. The absence of the ~1743 cm^−1^ ester carbonyl band [112] in R1.5 may suggest altered lipid–protein association at this specific formulation, possibly reflecting differential partitioning of soy lipids when a small proportion of soybean is replaced by TMP.

Overall, the strength of hydrogen bonds and β-sheet network tends to reduce when TMP concentration increases and soy protein concentration decreases. This is likely a consequence of soybean protein (11S, 7S) dilution and phase separation, which is commonly observed in protein/polysaccharides mixed systems in food processing due to thermodynamic incompatibility of the polymers [113].

### 4.2. Effects on Rheological Behaviour

According to Table A1, Appendix A, from the onset of rheological measurement, G′ exceeded G″ for all treatments, confirming that CaSO_4_ immediately induced elastic network formation through electrostatic interactions and salt bridge formation between denatured soybean protein molecules in the presence of Ca^2+^ [114,115]. Notably, compared with the control, higher TMP concentration in enriched soymilk (R5, R3) exhibited substantially higher initial G′ and G” values, and all TMP treatments showed significantly lower initial tanδ values, suggesting that TMP enhanced early-stage aggregation. This effect is contributed by the additional hydrogen bonds between TMP polysaccharides β-glucan hydroxyl groups and soy proteins during the initial aggregation phase, as reflected in O–H region of TMP tofu (A1.5, R1.5)’s FT-IR spectra. The replacement treatments exhibited significantly higher G′ and G″ values than the addition treatments during early gelation, suggesting that mushroom polysaccharides contributed more strongly to network formation when soy protein content was reduced. This may be because lower protein levels decreased protein–protein dominance between soy globulins 7S and 11S (which are key protein components forming tofu), allowing polysaccharide chains to interact through hydrogen bonding and physical entanglement. In contrast, the higher protein content in the addition treatments may have masked the rheological contribution of the polysaccharides.

Similar effects have been reported in other protein–polysaccharide systems, where β-glucans promoted early aggregate formation and improved gel elasticity at suitable concentrations by cross-linking with proteins, physically embedding in protein network, increasing hydrogen bonding and electrostatic interaction [116,117,118]. It was also proposed that β-glucan absorbs water at the beginning of heating, allowing protein molecules to come closer to aggregate and elevating viscosity of the food system [117]. Another possible mechanism for the increased initial gelling properties in higher TMP treatment is the formation of complexes between soy protein and chitin from mushroom cell wall. Previous studies have shown that soy protein fibrils and chitin can self-assemble driven by electrostatic attraction between their oppositely charged groups, enhancing rigidity of soy protein gel [119] and chitin microfibres may have served as a supporting framework to strengthen the structure of β-conglycinin (7S) gel [120]. This attraction between oppositely charged biopolymers in a protein–polysaccharide mixture is regarded as associative phase separation, resulting in solvent-rich and polymer-rich phases [121].

As heating progressed to 75 °C, the control reached the highest final G′ and G”, indicating that heat-induced soy protein denaturation became the dominant factor governing gel strengthening. Thermal unfolding of β-conglycinin and glycinin exposes hydrophobic regions and sulfhydryl groups, promoting hydrophobic association and disulfide bond formation, which are major contributors to the load-bearing structure of tofu gels [122,123]. The magnitude of these interactions is closely correlated with the extent of soy protein denaturation [93]. Consistent with this mechanism, it was reported that hydrophobic and electrostatic interactions, rather than hydrogen bonding, are the principal contributors to heat-induced soy protein isolate–mushroom (*Tremella fuciformis*) polysaccharide composite gels [124].

Within both the addition and replacement series, G′ and G″ decreased progressively with increasing TMP content, suggesting that excess polysaccharide interferes with the development of the protein network at elevated temperature. Above 5% TMP, this disruption became pronounced, likely reflecting dilution of the continuous protein phase and steric hindrance of protein–protein contacts required for hydrophobic association and disulfide formation. At low TMP levels (≤3%), the replacement treatments retained G′ and G″ values closer to the control than the corresponding addition treatments, indicating that the polysaccharide-to-protein ratio—rather than the absolute polysaccharide concentration—governs whether the mixed system maintains an effective gel structure. Protein–polysaccharide complexation is strongest at the optimal mixing ratio at which the opposite charges on the protein and polysaccharide are fully neutralised [125]. This is consistent with segregative phase-separation behaviour, in which proteins and polysaccharides have similar net charges, causing electrostatic repulsion and separation into protein-rich and polysaccharide-rich phases. A balanced biopolymer ratio concentrates protein within the protein-rich phase and reinforces the network, whereas an imbalanced ratio promotes thermodynamic incompatibility and weakens the gel [113,126]. In the protein-rich phase, soy proteins become more concentrated, increasing protein–protein proximity and promoting the formation of Ca^2+^-mediated bridges that support tofu network development.

During the cooling phase to 10 °C, all treatments exhibited a marked and rapid increase in both G′ and G″, reflecting further strengthening of the gel network after thermal aggregation. The greater increase in G′ than G″ suggests preferential reinforcement of the elastic component, resulting in a more rigid and connected network. Cooling may stabilise the structure by reducing molecular mobility, allowing rearrangement of tofu’s gel structure. A similar cooling effect on tofu using different coagulants was reported by Zhao et al. [95]. Additionally, low temperature may have enhanced hydrogen bond formation, which introduces additional physical cross-links within the pre-formed matrix [127]. Furthermore, electrostatic repulsion can reduce at low temperature due to a drop in kinetic energy when thermal energy reduces, allowing intensification of hydrogen bonding, hydrophobic interactions, and disulfide bonding [128]. Between different treatments, despite their differences in increasing rate of G’ and G”, the ranking was generally preserved from the previous heating phase (Control> R1.5 > R3 > A1.5, A3 >R5, A5), suggesting that cooling mainly developed and reinforced the existing protein network rather than forming new complexes.

The frequency sweep results further confirmed that all formulations behaved as viscoelastic gels, with G′ remaining higher than G″ across the tested frequency range. The moderate frequency dependence observed for most treatments is characteristic of physically cross-linked soy gels stabilised mainly by non-covalent interactions rather than permanent covalent bonds [129].

Notably, R1.5 exhibited viscoelastic behaviour closely comparable to the control, indicating that replacement of 1.5% soybean with TMP did not compromise gel integrity, suggesting that favourable TMP–protein interactions compensated for the slight reduction in soy protein. Similar preservation of gel rheology at low polysaccharide incorporation levels has been reported in soy protein systems [130,131].

### 4.3. TMP Enrichment’s Influences on Microstructure, Texture, and Physicochemical Attributes

The superior G′ and G″ of the control tofu during heating and cooling corresponded to a well-developed and uniform protein network seen in the control tofu’s CLSM image. This is characteristic of well-formed protein network of CaSO_4_-induced soy gel from fully denatured soy protein [93]. Consistent with the trend observed in rheological analysis, R1.5 showed a slightly coarser network. As TMP concentration increased, other treatments displayed disrupted networks with enlarged protein-depleted void regions beside protein clusters, especially in the R5 sample. This morphology reflects segregative phase separation suggested by rheology analysis. Jin, et al. [132] also reported that the microstructure of soy protein network is defined by the competition between gelation kinetics of gelling proteins and protein-polysaccharide phase separation. Furthermore, TMP incorporation could lead to a protein dilution effect reducing the effective concentration of gel-forming soy globulins.

Regarding texture profile, hardness reflects the force required to deform or break the tofu structure, while springiness describes its ability to recover after compression. Cohesiveness indicates the strength of internal bonding within the tofu matrix, and chewiness represents the energy required to chew the tofu until it is ready for swallowing [57]. Control tofu showed superior texture profile. In contrast, because of protein network disruption shown by CLSM images, there was a progressive decline in hardness, cohesiveness, and chewiness with increasing TMP concentration. It was reported that hardness of tofu depends critically on protein concentration, subunit composition. For example, glycinin (11S)-rich soymilk forms harder gels than β-conglycinin (7S)-rich soymilk. [86]. Although the protein-fraction profile of TMP was not determined in the present study, previous reports have shown relatively low globulin contents in several edible mushrooms, including Macedonian mushrooms (12.07% total protein) [133], Brazilian-cultivated oyster mushrooms (~3% protein extracts) [134], and *Cordyceps militaris* (~18% total protein) [135], whereas globulins account for about 90% of soy protein [85]. As tofu gelation mainly relies on soybean globulins 7S and 11S, TMP may provide insufficient compatible globulin-type proteins for network formation while simultaneously diluting the effective concentration of soy gel-forming proteins, thereby reducing tofu hardness. Likewise, Ullah et al. [136] also demonstrated that okara fibre addition to tofu continuously decreased hardness, springiness, cohesiveness, and chewiness because hydroxyl groups (-OH) on fibre molecules interfered with the gel-forming hydrophobic interactions between soy protein molecules, resulting in a disrupted network with reduced textural strength. Furthermore, particle size of TMP and its interaction with the gel matrix may also influence texture outcomes. Finer okara particles (<180 μm) produced tofu with higher gel strength, chewiness, and sensory scores than coarser particles because smaller particles integrate more readily into the protein network without creating large voids [137,138]. These findings suggest that future work in optimising TMP particle size could partially mitigate the textural losses observed at higher incorporation levels.

Aside from texture, yield is a critical quality parameter determining production efficiency [139]. Homogeneity and density of tofu’s protein network strongly correlates with tofu’s texture profile and yield [140]. Hence, consistent with the networks shown by CLSM images, control tofu with a continuous protein network had the best yield (in both wet and dry basis) whereas treatments with higher TMP levels showed coarser network and had lower yields. R1.5 had relatively higher yield (in wet and dry basis) than other TMP treatments, reflecting the superior network formation and gelling properties shown in rheological analysis. Wet yield is strongly influenced by water holding capacity which is the ability of a tofu gel to retain water within its protein network during external stress, such as centrifugation or pressing. A compact and homogeneous tofu network can immobilise more water and soybean solids, thereby improving wet yield, whereas a coarse or disrupted network promotes water loss and reduces yield [141,142]. Tofu yield is also influenced by profile of storage soy proteins 11S, 7S, and their ratio [139]. At lower concentration, termite mushroom’s polysaccharides, especially β-glucan in its cell wall [143], may enhance water-holding capacity by forming hydrogen bonds with water through its extensive hydrophilic hydroxyl (-OH) groups [104]. However, at higher concentrations (5% TMP), the progressive disruption of the soy protein network creates larger pores and voids that cannot physically retain water during pressing, resulting in serum expulsion and reduced WHC. This trend of WHC increased at low percentage and decreased at higher percentage of additives is similar to Ullah, et al. [144]’s study in okara fibre-fortified tofu. On the other hand, cooking loss is mainly governed by the thermal stability of the tofu gel network and its ability to retain water and soluble solids during heating [145]. Although WHC differed among treatments, the similar cooking loss suggests that TMP incorporation affected water retention under mechanical stress but did not markedly compromise the thermal stability of the tofu matrix during cooking.

### 4.4. Nutritional Implications of TMP Fortification

Despite the structural changes described above, all TMP-fortified tofu treatments retained crude protein contents above 47% on a dry basis, confirming that the product remains a high-protein food suitable for plant-based diets. Nonetheless, TMP-enriched tofu still showed a reduced protein content at higher TMP level (R5), which is most likely due to its reduced protein network discussed above. Interestingly, measurement of materials used in the present study showed that TMP itself contained 39.65 ± 0.56% protein (dry weight), higher than the value of soybean (35.62 ± 0.35%). Therefore, the addition of a protein-rich ingredient does not necessarily increase the final protein content unless its proteins are effectively integrated and retained within the food matrix, as illustrated in cheese systems where soluble proteins are otherwise lost during processing [146].

Edible mushrooms are typically a good source of essential minerals [147], which was reflected in the relatively high mineral content in treatment with more TMP such as R5. Iron exhibited the most nutritionally significant and consistent response to TMP fortification with R5’s content being over 30% higher than the control. It was measured that in this study, TMP had 52.76 ± 1.53 mg/kg Fe while soybean had a much lower Fe level at 11.44 ± 0.92 mg/kg dry weight. This iron richness in termite mushroom is consistent with other mushrooms documented, containing about 30–150 mg/kg Fe [148,149]. Regula, et al. [150] reported that cereals fortified with shiitake mushrooms had increased Fe content and were able to alleviate iron deficiency in female rats. As iron deficiency is a great concern for vegetarians [151], who are the primary consumer demographic for tofu, this iron enrichment represents a meaningful nutritional advantage of TMP fortification. Thus, TMP fortification preserves the essential nutritional characteristic of soy tofu while delivering a significant iron enrichment, representing a potentially favourable approach for nutritional optimisation.

Sodium content varied markedly but did not follow TMP concentration, suggesting that sodium retention was governed more by gel structure than by ingredient composition. Because Na^+^ is mainly associated with the aqueous phase, a coarser and more permeable protein network may have allowed greater loss of Na-containing serum during pressing, thereby reducing sodium retention in the final tofu. Ion interactions may have further contributed to this non-linear pattern, as Ca^2+^ binds more strongly than Na^+^ to negatively charged protein groups and can alter the distribution of soluble cations within the gel matrix. Similar effects of salt partitioning into expelled whey and cation interactions during syneresis have been reported in renneted casein gels [152], supporting the interpretation that Na variation reflects matrix retention and whey loss rather than TMP level alone.

### 4.5. Effects of TMP Incorporation and Cooking on Colour Changes

According to established colour science thresholds, ΔE values above 3.0–5.0 are considered clearly perceptible to the human eye [153], confirming that cooking-induced colour changes were visually significant in all formulations. The progressive darkening and increased chromaticity of TMP-containing tofu can be attributed to multiple mechanisms. *Termitomyces* spp. contain natural pigments including melanin and phenolic compounds that contribute directly to dark colour [154]. During cooking, additional browning occurs through thermally driven Maillard reactions between reducing sugars (contributed by mushroom carbohydrates) and amino acids, producing melanoidin, which are visible-light-absorbing chromophores [155]. Furthermore, polyphenol oxidase-catalysed oxidation of mushroom-derived phenolic substrates generates ortho-quinones that polymerise into brown pigments [156]. The increased redness and yellowness of TMP-tofu may be contributed by other common mushroom pigments such as carotenoids (β-carotene, lycopene) or anthraquinones [157].

From a product development perspective, the darker colour of TMP-fortified tofu may limit applications where a white appearance is preferred (e.g., traditional silken tofu), but could be advantageous for flavoured, marinated, or grilled tofu products where darker, richer colours are commercially acceptable or desirable. Similar dose-dependent darkening has been consistently reported when mushroom powder is incorporated into bread [158], pasta [159], and other bakery products [160].

### 4.6. Antioxidant Activity and Bioaccessibility During Simulated Gastrointestinal Digestion

#### 4.6.1. Chemical Basis and Selectivity of the Four Assays

The antioxidant properties of TMP-fortified tofu were evaluated using Folin–Ciocalteu total phenolic content assay (TPC), ABTS, DPPH, and FRAP assays because these in vitro antioxidant assays differ substantially in their chemical principles, strengths, and limitations [161]. Therefore, relying on one assay alone may overestimate or underestimate antioxidant potential, particularly in a mixed protein–polysaccharide–phenolic matrix such as mushroom-fortified tofu.

The Folin–Ciocalteu TPC assay provides an estimate of phenolic-associated reduction of a phosphomolybdate–phosphotungstate complex [162,163]. ABTS and DPPH both assess radical-scavenging capacity, but ABTS•^+^ is reactive in both aqueous and organic environments and is responsive to hydrophilic and lipophilic antioxidants, whereas DPPH• is more restricted to organic systems [164]. FRAP reflects the electron-donating capacity of antioxidants [165]. Together, these assays provide complementary information on different aspects of antioxidant potential.

#### 4.6.2. Antioxidant Potential of TMP-Fortified Tofu and Effects of Cooking

Overall, in fresh tofu, the higher level of TMP incorporation generally improved the antioxidant profile of the products in TPC, DPPH, and ABTS assays, despite no significant difference found in FRAP. This enhancing effect is due to termite mushroom’s high potential in antioxidant activities with its bioactive phenolic compounds and polysaccharides [36,166]. In a study testing *Termitomyces heimii* extracts, lower antioxidant results in DPPH and FRAP assays compared to other assays (hydroxyl radical scavenging assay, superoxide radical scavenging assay, total antioxidant capacity assay) were also reported [167]. This suggests DPPH and FRAP assays may not reflect the mechanisms of termite mushroom’s antioxidant activities whereas they were more detectable by the basis of single-electron-transfer of Folin and ABTS methods. The replacement series showed higher TPC than the addition series, though this was not clear for DPPH and ABTS assays. R5 generally held the highest values across the assays. This pattern is most plausibly explained by two complementary mechanisms. First, TMP likely contributed additional phenolic compounds directly to the tofu matrix as *Termitomyces* mushrooms are good sources of polyphenols [166]. Second, replacing part of the soybean fraction with TMP reduced the amount of soy protein available to bind and sequester phenolics. Since phenolics can associate with soy proteins through non-covalent interactions, forming protein–phenolic complexes that limit phenolic release [168], the lower protein content in the R-series may have increased the proportion of extractable phenolics. For example, soybean β-conglycinin (7S) and glycinin (11S) can bind to flavonoids or cyanidin-3-O-glucoside by hydrogen bonding through phenolic hydroxyl (-OH) groups, and hydrophobic interactions, van der Waals forces with benzene rings and amino-acid residues with binding strength depending on phenolic structure [169,170].

The increase in TPC after cooking across all treatments suggests improved extractability of reducing compounds from the tofu matrix. Heating can disrupt structural polysaccharides, including the chitin–β-glucan matrix of TMP, thereby releasing insoluble-bound phenolics associated with cell wall components [171,172]. High temperature cooking may also inactivate polyphenol oxidase, the enzyme involved in enzymatic browning and phenolic oxidation in mushrooms, preventing further enzymatic loss of free phenolics [156]. In addition, the Folin–Ciocalteu assay is not phenolic-specific, so Maillard-derived reducing compounds formed during heating may also contribute to the higher apparent TPC [173]. Similar cooking effects were observed in ABTS assays but not DPPH and FRAP, indicating that the latter two assays are not efficient in accessing phenolics bound within the protein–polysaccharide tofu matrix or the water-soluble Maillard polymers [174,175]. The trend of higher TMP, higher antioxidant activities was generally preserved after frying.

#### 4.6.3. Phase-Dependent Antioxidant Release During Simulated Digestion

There was a consistent pattern of progressively increasing antioxidant signal from oral → gastric → intestinal phases, accompanied by marked divergence among the four assays. Polyphenols and other antioxidant ingredients in food are not always present in a freely extractable form, they can be partially retained in the network of proteins, carbohydrates, dietary fibre, and other nutrients. Non-covalent protein–polyphenol complexes can limit phenolic extractability; however, hydrolysis during digestion may disrupt these associations and release bound polyphenols [176]. Mechanistically, each transition reflects different aspects of matrix breakdown and antioxidant release. Despite minor fluctuations, TMP treatments also consistently showed higher antioxidant activities, especially at high TMP concentration of R5 treatment.

The uniformly low antioxidant values in the oral phase indicate limited release of active compounds at this stage. This is expected because oral digestion is brief and salivary α-amylase acts mainly on starch rather than on the protein–polysaccharide interactions that retain antioxidants within the tofu matrix [67]. The static INFOGEST model also does not fully reproduce mastication, which may further limit matrix disruption. The unusually high oral-phase DPPH value in R1.5 may therefore reflect a greater proportion of readily soluble or weakly bound mushroom-derived antioxidants in this formulation, such as low-molecular-weight phenolics located closer to the gel surface [36]. However, this pattern was not consistent across assays.

The rise in TPC and especially DPPH during the gastric phase indicates partial liberation of matrix-bound antioxidants under acidic and proteolytic conditions. Pepsin digestion can disrupt soy protein–phenolic complexes, while the acidic environment (pH 3.0) weakens hydrogen bonding and electrostatic interactions that retain phenolics within the CaSO_4_-induced tofu gel network [177]. Hydrolysis progressively disrupts protein–phenolic complexes, restoring accessibility of antioxidant functional groups previously masked within the matrix [178]. The stronger gastric response in DPPH than in TPC further suggests that digestion altered not only the amount of extractable reducing material but also the availability of radical-scavenging compounds.

The intestinal phase was the major stage of antioxidant bioaccessibility, as shown by the strong increases in ABTS and FRAP and the recovery of TPC. This likely reflects more extensive matrix breakdown by pancreatin, which can further hydrolyse residual protein and polysaccharide structures remaining after gastric digestion [179]. Bile salts may also enhance the dispersion and solubilisation of relatively hydrophobic antioxidant compounds including soy isoflavone aglycones (genistein, daidzein, glycitein), tocopherols, and mushroom-derived terpenoids, bringing them into the aqueous phase where they become accessible to the spectrophotometric assays [180]. In addition, neutral intestinal pH may promote ionisation of phenolic hydroxyl groups and favour electron transfer reactions, contributing to the strong intestinal-phase response [181].

The divergence among assays reflects their different chemical bases. ABTS showed the largest and most uniform increase, consistent with its broad responsiveness to both hydrophilic and lipophilic antioxidants [182]. FRAP remained low until intestinal digestion, indicating that ferric-reducing compounds became measurable only after substantial matrix disintegration [163]. DPPH showed the greatest treatment-dependent variability, likely because it is more sensitive to specific radical-scavenging compounds released from each formulation in methanolic medium [163]. The anomalously low intestinal DPPH value of R5 may be explained by degradation or transformation of specific DPPH-reactive antioxidants at the near-neutral intestinal pH, in the presence of bile salts, pancreatin, and digestion-derived peptides [177]. They can lose the single H-atom/electron-transfer capacity that the lipophilic, ethanol-based DPPH assay detects, while still registering in the aqueous-compatible ABTS and FRAP assays [183]. The known sensitivity of DPPH to matrix interference and to hydrophilic antioxidants is consistent with the divergence between assays in this study [184]. These differences support the use of multiple assays when assessing antioxidant bioaccessibility [185].

Although intestinal ABTS and FRAP values were broadly similar among treatments, TPC and especially DPPH remained formulation-dependent. This suggests that TMP affected not only antioxidant content but also the retention and release of antioxidants within the tofu matrix. The strong intestinal DPPH response of R1.5 suggests that moderate TMP replacement may favour antioxidant bioaccessibility, whereas lower values in some treatments may reflect phenolic oxidation during processing or formation of less digestible phenolic–protein aggregates [186]. Mushroom components may also contribute directly to this response, including β-glucan-associated bioactive release and the presence of the stable mushroom antioxidant ergothioneine [187,188].

## 5. Conclusions

This study demonstrated that whole *Termitomyces albuminosus* mushroom freeze-dried powder (TMP) is a promising functional ingredient for soy tofu, producing dose- and method-dependent changes in its structure, rheology, nutritional composition, and antioxidant properties. While the basic tofu gel structure was preserved, increasing TMP incorporation progressively weakened the protein network, altered the microstructural organisation, thereby influencing tofu quality attributes (water holding capacity, colour, texture). However, at a low level of 1.5% TMP replacing soybean (R1.5), gel structure, protein network and texture, and water holding capacity were preserved. TMP fortification improved mineral content, particularly iron and calcium, without compromising overall protein adequacy, and enhanced antioxidant bioaccessibility during digestion, especially in the intestinal phase. Among the tested formulations, tofu with 5% soybean replaced by termite mushroom powder (R5) showed the greatest nutritional improvement, with the highest phenolic and ABTS values, despite its disrupted texture. Therefore, in TMP-fortified tofu, higher fortification is not necessarily superior, as it involves a trade-off between improved nutritional functionality and the preservation of structural quality. In this regard, the R1.5 formulation showed greater practical potential by relatively preserving tofu quality while still improving nutritional value. Future applications should optimise the polysaccharide–protein balance to maximise nutritional functionality while preserving gel structure, textural quality, and consumer acceptability. Of the two fortification strategies: direct addition and soybean replacement, the latter showed more pronounced effects on both quality and nutritional attributes. These results highlight the potential of *T. albuminosus*-fortified tofu as a value-added, plant-based product with enhanced functional and nutritional attributes. For commercial-scale application, further studies should assess the viability and market potential of mushroom-fortified tofu through cost–benefit analysis, consumer-demand evaluation, and sensory testing, the absence of which is acknowledged as a limitation of this study. Future research should also focus on formulation optimisation, particularly improving texture, colour, storage stability, and shelf life, while directly quantifying the bioaccessibility of key functional components, including β-glucans, chitin, and phenolics, to clarify the mechanisms underlying the observed nutritional and antioxidant benefits.

## Figures and Tables

**Table 1:** Yield and quality characteristics of tofu enriched with TMP.

Treatment	Wet-Basis Yield (%)	Dry-Basis Yield (%)	Moisture (%)	pH	Water Activity (a_w_)	Water Holding Capacity (%)	Cook Loss (%)
Control	177.53 ± 16.97 ^a^	50.75 ± 0.25 ^a^	73.41 ± 2.28 ^a^	6.49 ± 0.09 ^a^	≈1.00	80.08 ± 4.77 ^a,b,c^	29.28 ± 4.2 ^a^
A1.5	134.71 ± 15.75 ^b^	40.51 ± 5.53 ^a,b^	73.24 ± 1.92 ^a^	6.46 ± 0.13 ^a^	≈1.00	78.15 ± 3.54 ^b,c^	28.1 ± 0.29 ^a^
A3	115.00 ± 5.08 ^b^	35.98 ± 3.01 ^b^	72.13 ± 2.07 ^a^	6.4 ± 0.13 ^a^	≈1.00	83.04 ± 6.58 ^a,b^	26.65 ± 0.87 ^a^
A5	125.92 ± 9.11 ^b^	34.6 ± 6.1 ^b^	75.64 ± 2.32 ^a^	6.41 ± 0.05 ^a^	≈1.00	75.78 ± 5.48 ^b,c^	25.62 ± 0.49 ^a^
R1.5	141.51 ± 17.59 ^a,b^	44.16 ± 3.86 ^a,b^	72.11 ± 1.8 ^a^	6.45 ± 0.07 ^a^	≈1.00	87.80 ± 2.9 ^a^	18.44 ± 4.08 ^a^
R3	141.51 ± 17.59 ^a,b^	38.38 ± 5.65 ^a,b^	72.57 ± 3.67 ^a^	6.39 ± 0.15 ^a^	≈1.00	82.51 ± 6.42 ^a,b^	25.95 ± 4.99 ^a^
R5	132.51 ± 6.27 ^b^	39.57 ± 4.97 ^a,b^	73.38 ± 3.64 ^a^	6.39 ± 0.07 ^a^	≈1.00	72.02 ± 2.19 ^c^	26.60 ± 1.09 ^a^

Different superscript letters (a, b, c) within the same column indicate significant differences among mean values (*p* < 0.05). Control: 100% soybean, A1.5: additional 1.5% TMP, A3: additional 3% TMP, A5: additional 5% TMP, R1.5: 1.5% soybean weight replaced by TMP, R3: 3% soybean weight replaced by TMP, R5: 5% soybean weight replaced by TMP.

**Table 2:** Colour indices L*, a*, b* of fresh and cooked tofu and changes of colour after cooking (ΔL*, Δa*, Δb*, ΔE).

Treatment	Fresh Tofu			Cooked Tofu			Cooking-Induced Colour Change			
	L*	a*	b*	L*	a*	b*	∆L^∗^	∆a^∗^	∆b^∗^	∆E
Control	79.14 ± 0.35 ^a^	−0.05 ± 0.1 ^d^	14.01 ± 0.36 ^a,b^	74.02 ± 1.65 ^a^	1.82 ± 0.57 ^d^	17.09 ± 3.17 ^b^	−5.12 ± 1.73 ^d^	1.86 ± 0.57 ^b^	3.09 ± 3.28 ^b^	7.04 ± 0.45 ^d^
A1.5	76.99 ± 0.08 ^b^	0.68 ± 0.04 ^b^	12.03 ± 0.79 ^d^	69.54 ± 0.42 ^b^	3 ± 0.17 ^b^	21.15 ± 1.43 ^a^	−7.45 ± 0.48 ^c,d^	2.32 ± 0.18 ^b^	9.12 ± 0.86 ^a^	12 ± 0.94 ^b^
A3	76.4 ± 0.55 ^b,c^	0.9 ± 0.15 ^a,b^	13.11 ± 0.81 ^a,b,c,d^	63.42 ± 1.16 ^d^	4.57 ± 0.28 ^a^	20.09 ± 1.52 ^a,b^	−12.99 ± 1.4 ^a^	3.66 ± 0.22 ^a^	6.98 ± 1.81 ^a,b^	15.26 ± 1.6 ^a^
A5	75.65 ± 0.88 ^c^	0.75 ± 0.02 ^b^	12.9 ± 0.71 ^b,c,d^	65.05 ± 1.41 ^c,d^	2.83 ± 0.1 ^b,c^	17.37 ± 1.02 ^a,b^	−10.6 ± 0.83 ^a,b,c^	2.09 ± 0.1 ^b^	4.48 ± 0.99 ^b^	11.74 ± 0.37 ^b,c^
R1.5	78.19 ± 0.59 ^a^	0.28 ± 0.09 ^c^	14.44 ± 0.3 ^a^	69.94 ± 0.16 ^b^	2.23 ± 0.04 ^c,d^	17.98 ± 1.24 ^a,b^	−8.25 ± 0.61 ^a,b,c^	1.95 ± 0.09 ^b^	3.54 ± 1.54 ^b^	9.25 ± 1 ^c,d^
R3	76.51 ± 0.26 ^b,c^	1.05 ± 0.18 ^a^	13.76 ± 0.22 ^a,b,c^	67.63 ± 2.1 ^b,c^	2.97 ± 0.48 ^b^	18.04 ± 1.4 ^a,b^	−8.89 ± 2.2 ^b,c^	1.92 ± 0.64 ^b^	4.28 ± 1.55 ^b^	10.28 ± 1.19 ^b,c^
R5	74.15 ± 0.30 ^d^	0.77 ± 0.07 ^b^	12.64 ± 0.57 ^c,d^	63.12 ± 1.6 ^d^	3.07 ± 0.21 ^b^	16.44 ± 1.05 ^a^	−11.03 ± 1.46 ^a,b^	2.3 ± 0.14 ^b^	3.8 ± 0.63 ^b^	11.9 ± 1.56 ^b^

Different superscript letters (a, b, c, d) within the same column indicate significant differences among mean values (*p* < 0.05).

**Table 3:** Proximate content of protein, ash, and mineral on tofu dry basis.

Treatment	Control	A1.5	A3	A5	R1.5	R3	R5
Protein (%) d.b.	57.79 ± 5.94 ^a^	47.81 ± 1.87 ^b^	55.15 ± 4.48 ^a,b^	52.13 ± 1.5 ^a,b^	51.57 ± 3.43 ^a,b^	53 ± 5.95 ^a,b^	48.24 ± 5.83 ^b^
Ash (%) d.b.	6.77 ± 0.87 ^a^	6.82 ± 0.67 ^a^	7.32 ± 0.6 ^a^	7.19 ± 0.46 ^a^	6.97 ± 1.13 ^a^	7.26 ± 0.84 ^a^	7.32 ± 0.8 ^a^
Na (mg/kg) d.b.	852.47 ± 112.3 ^a^	888.82 ± 69.72 ^a^	211.55 ± 2.06 ^c^	234.89 ± 6.01 ^c^	212.01 ± 11.38 ^c^	256.67 ± 73.5 ^c^	501.09 ± 64.56 b
P (mg/kg) d.b.	891.52 ± 176.26 ^a^	830.99 ± 78.71 ^a^	970.49 ± 63.78 ^a^	1053.61 ± 62.88 ^a^	1047.04 ± 67.74 ^a^	1031.58 ± 61.3 ^a^	831.49 ± 194.87 ^a^
K (mg/kg) d.b.	399.15 ± 86.71 ^a,b,c^	287.93 ± 28.01 ^c^	378.47 ± 19.11 ^a,b,c^	424.71 ± 15.49 ^a,b,c^	354.17 ± 41.74 ^b,c^	500.5 ± 53.35 ^a^	453.57 ± 123.23 ^a,b^
Mg (mg/kg) d.b.	177.34 ± 14.92 ^a^	148.57 ± 11.39 ^a^	183.16 ± 10.51 ^a^	202.37 ± 12.78 ^a^	186.07 ± 9.73 ^a^	185.47 ± 24.66 ^a^	201.95 ± 62.47 ^a^
Ca (mg/kg) d.b.	1812.43 ± 146.7 ^b,c^	1603.25 ± 121.79 ^c^	1710.95 ± 55.88 ^b,c^	1826.01 ± 87.76 ^b,c^	1936.09 ± 126.28 ^a,b^	1814.26 ± 118.57 ^b,c^	2177.8 ± 174.71 ^a^
Mn (mg/kg) d.b	2.87 ± 0.66 ^a^	2.58 ± 0.3 ^a^	3.15 ± 0.16 ^a^	3.65 ± 0.28 ^a^	3.22 ± 0.19 ^a^	3.25 ± 0.51 ^a^	3.62 ± 1.13 ^a^
Cu (mg/kg) d.b.	3.06 ± 0.2 ^a^	3.15 ± 0.28 ^a^	3.2 ± 0.16 ^a^	3.26 ± 0.33 ^a^	2.94 ± 0.09 ^a^	3.29 ± 0.46 ^a^	3.58 ± 0.75 ^a^
Fe (mg/kg) d.b.	20.61 ± 2.65 ^b,c^	19.49 ± 2.51 ^c^	21.02 ± 1.52 ^b,c^	26.13 ± 1.72 ^a,b^	20.58 ± 1.03 ^b,c^	25.44 ± 4.1 ^a,b^	27.07 ± 7.14 ^a^
Zn (mg/kg) d.b.	9.9 ± 1.87 ^a^	9.37 ± 1.15 ^a^	9.8 ± 0.56 ^a^	10.86 ± 0.72 ^a^	9.95 ± 0.4 ^a^	10.37 ± 1.57 ^a^	11.59 ± 3 ^a^
Al (mg/kg)d.b.	8.49 ± 0.82 ^a^	9.61 ± 0.75 ^a^	5.47 ± 0.38 ^c,d^	7.98 ± 1.44 ^b,c^	4.69 ± 0.35 ^d^	7.11 ± 1.1 ^b,c,d^	8.13 ± 2.65 ^b,c^

Different superscript letters within the same row indicate significant differences among mean values (*p* < 0.05). d.b. = dry basis.

**Table 4:** Texture profile parameters of tofu products.

Treatment	Hardness (g)	Springiness	Cohesiveness	Chewiness (g)
Control	1647.8 ± 130.1 ^a^	0.9994 ± 0.0001 ^a,b^	0.57 ± 0.03 ^a^	930.5 ± 32.3 ^a^
A1.5	743.4 ± 54.5 ^c^	0.9993 ± 0.0000 ^a,b^	0.37 ± 0.07 ^b^	272.8 ± 33.4 ^c^
A3	1286.7 ± 32.2 ^b^	0.9994 ± 0.0002 ^a,b^	0.38 ± 0.04 ^b^	494.0 ± 65.3 ^b^
A5	427.9 ± 24.2 ^d^	0.9993 ± 0.0002 ^a,b^	0.32 ± 0.03 ^b,c^	138.5 ± 12.4 ^d^
R1.5	841.9 ± 62.7 ^c^	0.9994 ± 0.0001 ^a,b^	0.39 ± 0.05 ^b^	323.6 ± 42.9 ^c^
R3	257.1 ± 38.1 ^d^	0.9992 ± 0.0000 ^b^	0.24 ± 0.04 ^c^	60.8 ± 13.2 ^e^
R5	200.8 ± 16.0 ^d^	0.9995 ± 0.0001 ^a^	0.26 ± 0.03 ^c^	52.1 ± 9.49 ^e^

Means with different superscript letters within a column are significantly different (*p* < 0.05).

**Table 5:** Antioxidant capacity of control and TMP-enriched tofu in fresh and cooked states and during in vitro digestion.

Total Phenolic Content (mg GAE/g)					
Treatment	Fresh Tofu	Cooked Tofu	Oral Digestion	Gastric Digestion	Intestine Digestion
Control	1.71 ± 0.2 ^Bb^	4.04 ± 0.29 ^Ab^	0.63 ± 0.11 ^Zb^	1.52 ± 0.33 ^Ya^	3.44 ± 0.05 ^Xb^
A1.5	2.81 ± 0.68 ^Bb^	5.6 ± 0.92 ^Aa,b^	0.74 ± 0.07 ^Ya,b^	1.97 ± 0.5 ^Ya^	4.27 ± 0.69 ^Xa,b^
A3	2.81 ± 0.68 ^Bb^	6.65 ± 1.44 ^Aa,b^	0.8 ± 0.06 ^Za,b^	1.49 ± 0.19 ^Ya^	3.84 ± 0.23 ^Xa,b^
A5	5.08 ± 0.41 ^Aa^	6.65 ± 1.44 ^Aa,b^	0.71 ± 0.01 ^Za,b^	1.74 ± 0.07 ^Ya^	3.48 ± 0.12 ^Xb^
R1.5	5.39 ± 0.3 ^Ba^	8.16 ± 0.46 ^Aa,b^	0.8 ± 0.06 ^Za,b^	2.17 ± 0.6 ^Ya^	4.16 ± 0.45 ^Xa,b^
R3	4.67 ± 0.41 ^Ba^	7.25 ± 0.02 ^Aa,b^	0.81 ± 0.06 ^Za^	1.89 ± 0.21 ^Ya^	3.89 ± 0.05 ^Xa,b^
R5	5.17 ± 0.9 ^Ba^	7.88 ± 1.42 ^Aa,b^	0.82 ± 0.05 ^Ya^	1.69 ± 0.33 ^Ya^	4.82 ± 0.79 ^Xa^
DPPH (µmol TEAC/g)					
Treatment	Fresh tofu	Cooked tofu	Oral digestion	Gastric digestion	Intestine digestion
Control	0.72 ± 0.05 ^Ac^	0.81 ± 0.06 ^Aa^	0.13 ± 0.12 ^Zd^	9.13 ± 2.19 ^Ya^	18.16 ± 5.49 ^Xb^
A1.5	0.95 ± 0.10 ^Aa,b,c^	0.94 ± 0.09 ^Aa^	1.70 ± 0.78 ^Yc,d^	8.14 ± 1.22 ^Ya^	3.38 ± 2.02 ^Xc^
A3	1.05 ± 0.03 ^Aa,b^	1.16 ± 0.37 ^Aa^	4.98 ± 1.15 ^Zb^	12 ± 1.78 ^Ya^	20.8 ± 2.34 ^Xa,b^
A5	1.06 ± 0.05 ^Aa,b^	1.17 ± 0.27 ^Aa^	1.91 ± 0.4 ^Yc,d^	9.74 ± 3.01 ^Xa^	10.34 ± 2.9 ^Xb,c^
R1.5	1.16 ± 0.02 ^Aa^	0.96 ± 0.46 ^Aa^	15.44 ± 1.63 ^Za^	9.08 ± 1.14 ^Ya^	29.44 ± 2.06 ^Xa^
R3	0.91 ± 0.11 ^Ab,c^	0.91 ± 0.22 ^Aa^	3.36 ± 1.4 ^Yb,c^	13.6 ± 5.31 ^X,Ya^	17.5 ± 7.53 ^Xb^
R5	0.97 ± 0.15 ^Aa,b,c^	0.77 ± 0.02 ^Aa^	0.74 ± 0.54 ^Xc,d^	6.49 ± 4.98 ^Xa^	2.36 ± 0.47 ^Xc^
ABTS (µmol TEAC/g)					
Treatment	Fresh tofu	Cooked tofu	Oral digestion	Gastric digestion	Intestine digestion
Control	2.46 ± 0.4 ^Bc^	3.94 ± 0.25 ^Ac^	26.78 ± 0.76 ^Yb^	19.12 ± 0.29 ^Yd^	158.01 ± 16.92 ^Xa^
A1.5	4.12 ± 0.35 ^Ba,b^	4.94 ± 0.24 ^Aa,b^	26.62 ± 0.63 ^Yb^	38.07 ± 1.27 ^Yc^	159.69 ± 10.69 ^Xa^
A3	3.72 ± 0.12 ^Bb^	4.56 ± 0.45 ^Aa,b,c^	26.28 ± 0.51 ^Zb^	42.79 ± 3.25 ^Yb,c^	162.72 ± 8.63 ^Xa^
A5	3.9 ± 0.12 ^Bb^	4.45 ± 0.07 ^Ab,c^	24.59 ± 4.9 ^Yb^	44.47 ± 1.34 ^Yb^	158.34 ± 20.77 ^Xa^
R1.5	4.1 ± 0.26 ^Aa,b^	4.28 ± 0.27 ^Ab,c^	29.73 ± 0.39 ^Za,b^	44.13 ± 2.05 ^Yb^	168.12 ± 3.55 ^Xa^
R3	3.79 ± 0.35 ^Bb^	4.83 ± 0.39 ^Aa,b^	29.82 ± 2.78 ^Ya,b^	47.84 ± 1.77 ^Ya,b^	140.49 ± 23.9 ^Xa^
R5	4.77 ± 0.15 ^Ba^	5.32 ± 0.2 ^Aa^	33.1 ± 0.25 ^Za^	51.04 ± 1.82 ^Ya^	169.8 ± 1.75 ^Xa^
FRAP (µmol TEAC/g)					
Treatment	Fresh tofu	Cooked tofu	Oral digestion	Gastric digestion	Intestine digestion
Control	1.27 ± 0.46 ^Ba^	2.25 ± 0.15 ^Aa,b^	0.56 ± 0.01 ^Ya^	0.52 ± 0.12 ^Ya^	11.05 ± 0.54 ^Xa^
A1.5	1.61 ± 0.44 ^Ba^	2.83 ± 0.28 ^Aa^	0.56 ± 0.08 ^Ya^	0.8 ± 0.22 ^Ya^	8.84 ± 0.02 ^Xb^
A3	1.32 ± 0.18 ^Ba^	2.49 ± 0.14 ^Aa,b^	0.51 ± 0.03 ^Ya^	0.85 ± 0.49 ^Ya^	10.69 ± 1.63 ^Xa,b^
A5	1.31 ± 0.15 ^Ba^	2.18 ± 0.4 ^Aa,b^	0.56 ± 0.11 ^Ya^	0.52 ± 0.11 ^Ya^	12.07 ± 0.8 ^Xa^
R1.5	1.95 ± 0.02 ^Aa^	1.86 ± 0.29 ^Ab^	0.57 ± 0.07 ^Ya^	0.62 ± 0.12 ^Ya^	11.19 ± 0.37 ^Xa^
R3	1.46 ± 0.24 ^Ba^	2.03 ± 0.23 ^Ab^	0.5 ± 0.04 ^Ya^	0.71 ± 0.24 ^Ya^	10.39 ± 0.23 ^Xa,b^
R5	1.74 ± 0.04 ^Aa^	2.05 ± 0.15 ^Ab^	0.56 ± 0.08 ^Ya^	0.64 ± 0.17 ^Ya^	11.33 ± 0.67 ^Xa^

Different superscript lowercase letters within the same column for each assay indicate significant differences among treatments (*p* < 0.05). Different superscript uppercase letters within the same row for each assay indicate significant differences between cooking states (A, B) and among digestion phases (X, Y, Z) (*p* < 0.05).

## Data Availability

The original contributions presented in this study are included in the article. Further inquiries can be directed to the corresponding author.

## Abbreviations

The following abbreviations are used in this manuscript:

TMP	Termite mushroom (freeze-dried) powder
TPA	Texture profile analysis
WHC	Water holding capacity
CLSM	Confocal laser scanning microscopy
FT-IR	Fourier transform infrared spectroscopy
TPC	Total phenolic content
FRAP	Ferric reducing antioxidant power (assay)
ABTS	2,2′-azino-bis(3-ethylbenzothiazoline-6-sulfonic acid
DPPH	2,2-diphenyl-1-picrylhydrazyl

## Appendix A

**Table A1:** Rheological properties of tofu products.

Treatment	Initial tanδ	Initial G′ (Pa)	Initial G″ (Pa)	G′ at 75 °C	G″ at 75 °C
Control	0.48 ± 0.09 a	7.91 ± 3.68 d	1.92 ± 1.11 b	239.05 ± 32.19 a	23.88 ± 5.38 a,b
A1.5	0.31 ± 0.04 b	31.23 ± 5.91 a,b,c	6.13 ± 3.1 a,b	172.95 ± 27.58 b	26.42 ± 12.63 a,b
A3	0.27 ± 0.01 b	26.09 ± 4.77 b,c	5.25 ± 1.55 a,b	157.7 ± 24.17 b,c	20.72 ± 3.85 a,b
A5	0.28 ± 0.01 b	19.69 ± 4.14 c,d	4.74 ± 1.32 a,b	116.14 ± 10.93 c,d	13.17 ± 3.45 b
R1.5	0.27 ± 0.01 b	28.18 ± 6.6 a,b,c	5.68 ± 2.51 a,b	238.89 ± 3.09 a	29.46 ± 2.58 a
R3	0.26 ± 0.02 b	40.22 ± 3.8 a	8.02 ± 4.09 a	210.81 ± 18.23 a,b	27.7 ± 0.65 a,b
R5	0.3 ± 0.04 b	37.03 ± 3.31 a,b	4.89 ± 4.68 a,b	97.94 ± 10.57 d	16.15 ± 0.18 a,b

Values are mean ± standard deviation. Means with different letters within a column are significant different (*p* < 0.05).
